# Assessing the Impact of Distance Traveled and Birth Volumes of Hospital Maternity Units on Newborn Outcomes: Population-Based Cohort Study

**DOI:** 10.2196/58944

**Published:** 2025-01-21

**Authors:** Anna Cantarutti, Riccardo Boracchini, Roberto Bellù, Raffaella Ronco, Federico Rea, Anna Locatelli, Rinaldo Zanini, Giovanni Corrao

**Affiliations:** 1 National Centre for Healthcare Research & Pharmacoepidemiology, University of Milano-Bicocca, , Milan, Italy; 2 Neonatal Intensive Care Unit, Woman and Child Health Department, Azienda Ospedaliera della Provincia di Lecco, Lecco, Italy; 3 Department of Obstetrics, IRCCS San Gerardo dei Tintori Foundation, University of Milano-Bicocca, Monza, Italy; 4 General Management, IRCCS Istituto Giannina Gaslini, Genova, Italy

**Keywords:** maternity level, road travel distance, delivery, regionalization, neonatal outcome, childbirth, newborn, cohort, birth, population-based, maternal, maternal outcomes, neonatal, European, health system, health care system, perinatal care, antenatal, mortality, neonatal mortality, perinatal

## Abstract

**Background:**

The centralization of childbirth and newborn care in large maternity units has become increasingly prevalent in Europe. While this trend offers potential benefits such as specialized care and improved outcomes, it can also lead to longer travel and waiting times, especially for women in rural areas.

**Objective:**

This study aimed to evaluate the association between hospital maternity unit (HMU) volumes, road travel distance (RTD) to the hospital, and other neonatal outcomes.

**Methods:**

We conducted a population-based cohort study including all live births in hospitals without intensive care units between 2016 and 2019 in the Lombardy region, Italy. Given the hierarchical structure of our data (births nested within hospitals), we employed log-binomial regression models with random intercepts to estimate relative risks and 95% CIs for evaluating the association between HMU volumes (≥1500 births/year) and RTD (<5 km) with the risk of being transferred and/or death after birth (primary outcome). Secondary outcomes included a low Apgar score at 5 minutes and low adherence to antenatal care (ANC). We controlled for several potential confounders including adherence to the ANC pathway for the primary and low Apgar outcomes. To explore the influence of HMU volumes on the primary outcome, we identified the fractional polynomial model that best described this relationship.

**Results:**

Of 65,083 live births, 71% (n=45,955) occurred in low-volume hospitals (<1000 births/year), 21% (n=13,560) involved long-distance travel (>15 km), 1% (n=735) were transferred and/or died after birth, 0.5% (n=305) had a low Apgar score at 5 minutes, and 64% (n=41,317) completely adhered to ANC. The risk of transfer and/or death increased as HMU volume decreased, ranging from 1% for hospitals with 1000‐1500 births/year to a 3.6-fold high risk for hospitals with <500 births/year (compared to high-volume hospitals). Travel distance did not affect the primary outcome. Neither HMU volume nor RTD were associated with low Apgar scores. Conversely, the risk of complete adherence to ANC decreased with lower HMU volumes but increased with shorter RTD. Additionally, high-volume hospitals demonstrated a decreasing trend in the frequency of the primary outcome, with transfer and/or death rates ranging from 2% to 0.5% and flattening to 0.5% in hospitals, with activity volume ≥1000 mean births/year.

**Conclusions:**

Our findings showed an excess risk of neonatal transfer and/or death for live births in HMUs with low activity volumes without an intensive care unit. In contrast, RTD primarily affected adherence to ANC. Moreover, data suggest that 1000 births/year could be an optimal cutoff for maternity hospitals to ensure an appropriate standard of care at delivery.

## Introduction

Driven by a commitment to improving maternal and neonatal outcomes, several European health care systems, including the Italian National Health Service (NHS) have embraced the regionalization of perinatal care in large maternity units since the 1980s. Perinatal regionalization aims to optimize access to quality care by organizing maternity and neonatal services into distinct levels based on the complexity of care required. This approach involves (1) ensuring that pregnant women and newborns are directed to the appropriate level of care based on their needs, (2) implementing early screening and assessment mechanisms to identify women at risk, and (3) facilitating timely and appropriate transfers between levels of care when necessary. By implementing these strategies, perinatal regionalization can improve outcomes for both mothers and infants while ensuring the efficient use of health care resources [1-4].

While perinatal regionalization offers potential benefits, its implementation remains debated. Robust, real-world evidence is needed to inform decision-makers about both the advantages of centralization (eg, reduced travel time, specialized care) and the potential disadvantages (eg, increased travel distance for some women). Moreover, only a few studies have comprehensively examined the combined effects of hospital maternity unit (HMU) volume and road travel distance (RTD) on maternal and neonatal outcomes [5-7]. However, the centralization of HMUs inevitably leads to the closure of some units and increases the distance (and travel time) to the hospital for some mothers.

Regionalization of perinatal care in HMUs with at least 1000 births/year has been undertaken since 2010 in Italy, although some maternity units continue to record fewer than 500 births/year. Despite the heterogenous geographical conformation of Italy, the distances between maternity units are relatively modest, compared to those reported by North American studies [8]. This finding combined with the ongoing efforts to regionalize HMU volumes to units with at least 1000 births/year, makes Italy an ideal setting to investigate the relationship between HMU volume, travel distance, and perinatal outcomes.

In this study, we conducted a population-based cohort study in Lombardy, the largest and most populous region of Italy, to investigate the singular and combined effects of HMU volumes and RTD on specific outcomes in births occurring in hospitals without intensive care units (ICUs). Additionally, we studied the influence of perinatal factors like antenatal care (ANC) and sociodemographic features of the mothers.

## Methods

### Data Source and Study Cohort

The study cohort consisted of all live infants born in Lombardy from January 1, 2016, to December 31, 2019. Lombardy is a region in Italy that accounts for approximately 16% of the country’s population, comprising nearly 10 million inhabitants. The health care utilization of all residents of Lombardy is covered by the government-funded NHS, which employs an automated system of databases to collect a variety of information. This system includes demographic and administrative data for all beneficiaries of the Regional Health Service (approximately covering the entire resident population), such as residence municipalities. Additional databases include the hospital discharges registry, which records all patients discharged from public or private hospitals; the outpatient drug prescription registry that reports all dispensations of NHS-reimbursable drugs; and the specialist visits and diagnostic exams registry including a specific automated system that collects data from the regional Department of Mental Health, accredited by the NHS and focused on outpatient specialist mental health care. Lastly, the Certificates of Delivery Assistance provide detailed information about pregnancy, childbirth, and fetal presentation at delivery. A unique deidentification code is systematically used for all databases; as a result, linking these records enables the creation of a large birth cohort and establishing relevant traits and care pathways for mothers and newborns.

**Figure 1.**
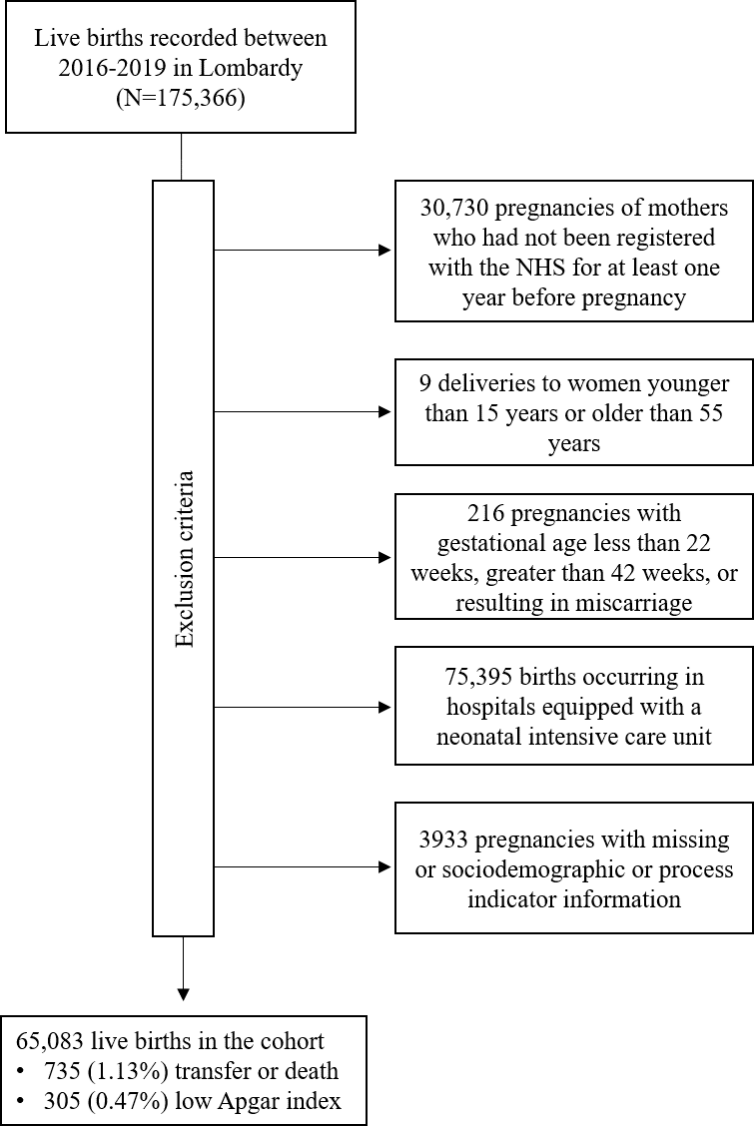
Flowchart of inclusion and exclusion criteria. NHS: National Health Service.

The criteria for selecting the study cohort almost completely overlapped with those previously reported by our group [9]. Briefly, using the Certificates of Delivery Assistance database, we identified all live births in Lombardy between 2016 and 2019 and available identification codes to women who met the inclusion criteria: (1) were beneficiaries of the NHS and had been residents of Lombardy for at least one year before pregnancy, (2) were aged 15 to 55 years at delivery, and (3) delivered between 22 to 42 weeks of gestation, based on the first day of the last menstrual period ascertained via maternal reports or ultrasonography. All births recorded in maternity hospitals equipped with an ICU were excluded. Further, records with incomplete data were excluded because baseline covariates such as sociodemographic and gestational information may be missing for some women, and limiting analyses to the subset of women for whom complete data were available would not result in a significant loss of information (Figure 1).

The results were reported in agreement with the STROBE (Strengthening the Reporting of Observational Studies in Epidemiology) statement guidelines [10].

### Categorization of HMU and RTD

The HMU volumes for each hospital were obtained based on the mean number of births recorded between 2016 and 2019. Categories were defined based on the activity volume of the hospital in which the birth occurred (ie, volumes ≤500 births/year, 500‐1000 births/year, 1000-1500 births/year, and >1500 births/year).

The RTD between the hospital and the mother’s residence municipalities was calculated according to the distance matrix (ie, an open-source distance matrix database provided by the Italian Institute of Statistics) [11]. Births were classified based on the mother having traveled <5 km, 5‐15 km, 15‐21.5 km, or ≥21.5 km to the HMU.

### Outcomes

The primary outcome of interest was transfer to a different hospital or death of the infant during the same birth hospitalization. The secondary outcomes included (1) a low Apgar score (<7) at 5 minutes after birth [12] and (2) complete adherence of the mothers to ANC during pregnancy. Maternal ANC adherence was evaluated based on the promptness and appropriateness of the number and timing of ANC interventions, including prenatal visits, ultrasound examinations, and laboratory tests, in relation to the length of pregnancy. In particular, the ANC assessment included (1) appropriateness of prenatal visits (ie, at least 5 visits during pregnancy or 2-4 visits for women with a gestational duration of <27 weeks); (2) promptness of prenatal visits (ie, at least 1 of the 4 visits within the 12th week of gestation); (3) appropriateness of ultrasound examinations (ie, ≥2 examinations during pregnancy, with at least 1 within the 12th week of gestation); and (4) appropriateness of laboratory tests, defined as completion of recommended tests for each trimester [13]. Women were identified as treatment-adherent if they followed all 4 of the recommendations.

### Definitions of the Covariates

Several baseline maternal characteristics were considered. Sociodemographic features included nationality, marital status, employment, and educational attainment (ie, low: ≤5 years, medium: 6‐13 years, and high: ≥14 years of study corresponding to primary education or none, lower or upper secondary education, and at least a bachelor’s degree, respectively). Clinical information was obtained from the inpatient hospital registry, the outpatient drug prescriptions registry, and the specialist visits and diagnostic exams registry. Maternal comorbidities, including psychiatric comorbidities, medical comorbidities, obstetric characteristics, and health care utilization measures (ie, number of hospitalizations and distinct prescription drugs used as general markers of comorbidity) [14] were evaluated. Concomitant medications (ie, nonsteroidal anti-inflammatory drugs, antidepressants, antiepileptics, psycholeptics, antihypertensive drugs, and antidiabetic drugs) were measured from 1 year before the last menstrual period through the 22nd week of gestation. Information regarding the ANC of mothers was measured during pregnancy.

### Ethical Considerations

According to the Italian Medicines Agency guidelines [15], retrospective studies that do not involve direct contact with patients do not need written consent to process personal data when they are used for research purposes. Thus, no ethics board review or approval was required.

To preserve privacy, each identification code was automatically anonymized, with only the Regional Health Authority having access to identifying information, which may be released upon request from judicial authorities to guarantee data deidentification.

### Statistical Analysis

The baseline maternal characteristics and distribution of the ANC received by mothers during pregnancy were described overall and stratified by the primary outcome. The *t* test, *χ*^2^ test, or Fisher exact test were used as appropriate. Moreover, the distribution of outcomes was expressed as absolute and relative percentages across HMU volumes and RTD categories. The Cochran-Armitage trend test was implemented for trend analysis.

As our data had a multilevel structure—with births (level 1) nested within hospitals (level 2)—log-binomial regression models with random intercepts were used to estimate relative risks (RRs) with 95% CIs for associations between HMU volumes (reference: ≥1500 births/year) and RTD (reference: RTD<5 km) and other outcomes of interest. The interaction between HMU volumes and RTD was also assessed. Models were adjusted for a disease score that was calculated based on the primary outcome, including all covariates listed above. For analyses in which ANC adherence was the outcome of interest, we excluded variables related to the ANC pathway from the disease score calculations.

A stratified analysis was performed by HMU volume, categorized as low (<1000 births/year) and high (≥1000 births/year) HMU volumes. Further, RTD was categorized as short (<15 km) and long RTD (≥15 km). Finally, to examine the influence of HMU volumes on the primary outcome (ie, transfer to a different hospital or death), a fractional polynomial model (FPM) was applied (first, second, or third degree), which described the relationship through a stepwise selection [16]. FPMs are particularly useful to preserve the continuous nature of the covariates in regression models while accounting for potential nonlinear relationships. Hospitals were the statistical units for this analysis.

All analyses were performed using the SAS (version 9.4; SAS Institute). *P* values <.05 were considered statistically significant.

## Results

Among the 175,366 live births recorded in Lombardy between January 1, 2016, and December 31, 2019, 30,730 births were excluded because the mothers had not received health care from the NHS for at least 1 year before pregnancy, while 9 were excluded because the mothers were younger than 15 or older than 55 years. Furthermore, 216 births were excluded because the gestational age at delivery was outside the range of 22 to 42 weeks, 75,395 births were excluded because they occurred in hospitals with an ICU, and 3933 births were excluded because complete sets of information about the mothers were not available (Figure 1). The final cohort consisted of 65,083 live births, of which 735 (1.13%) newborns were either transferred to another hospital (n=731) or died (n=4), 305 (0.47%) had a low Apgar score at 5 minutes after birth, and 41,317 (63.5%) fully adhered to ANC.

Mothers of newborns included in this cohort had a mean age of 32 (SD 5.4) years and mean gestational duration of 39 (SD 1.4) weeks. Most mothers had moderate educational attainment, were employed and married at delivery, and were of Italian nationality, and 55% (35,799/65,083) had already given birth. Maternal comorbidities were generally less prevalent and did not significantly differ across groups, except for psychoses and substance dependence, which were more common among mothers whose newborns experienced hospital transfer or death. Additionally, mothers in the transfer or death group showed higher rates of antiepileptic and antidiabetic drug use (Table 1).

**Table 1.** Sociodemographic, clinical, and pregnancy characteristics of mothers in Lombardy from 2016‐2019.

Cohort characteristics	Live births (N=65,083)	Individuals not transferred and/or experienced death (n=64,348)	Individuals transferred and/or experienced death (n=735)	*P* value^a^
Sociodemographic characteristics^b^				
Age (years), mean (SD)	32.27 (5.37)	32.27 (5.36)	32.17 (5.49)	.64
Educational attainment, years of study^c^, n (%)				<.001
≥14	19,036 (29.25)	18,859 (29.31)	177 (24.08)	
6‐13	29,720 (45.66)	29,385 (45.67)	335 (45.58)	
≤5	16,327 (25.09)	16,104 (25.03)	223 (30.34)	
Employed, n (%)				<.001
No	22,832 (35.08)	41,835 (65.01)	416 (56.60)	
Yes	42,251 (64.92)	22,513 (34.99)	319 (43.40)	
Married, n (%)				.99
No	24,695 (37.94)	39,932 (62.06)	456 (62.04)	
Yes	40,388 (62.06)	24,4416 (37.94)	279 (37.96)	
Italian nationality, n (%)				.01
No	18,109 (27.82)	46,474 (72.22)	500 (68.03)	
Yes	46,974 (72.18)	17,874 (27.78)	235 (31.97)	
Maternal comorbidities^d^, n (%)				
Depression and anxiety	394 (0.61)	387 (0.60)	7 (0.95)	.22
Preeclampsia	44 (0.07)	43 (0.07)	1 (0.14)	.39
Hypertension	90 (0.14)	87 (0.14)	3 (0.41)	.08
Diabetes	219 (0.34)	214 (0.33)	5 (0.68)	.10
Obesity or overweight	70 (0.11)	69 (0.11)	1 (0.14)	.55
Psychoses^e^	120 (0.18)	116 (0.18)	4 (0.54)	.048
Neuropathic, nonneuropathic, and other pain	140 (0.22)	139 (0.22)	1 (0.14)	>.99
Substance dependence	21 (0.03)	19 (0.03)	2 (0.27)	.02
Concomitant medication, n (%)				
Antidepressants	1395 (2.14)	1375 (2.14)	20 (2.72)	.28
Psycholeptics	159 (0.24)	155 (0.24)	4 (0.54)	.11
Antiepileptics	426 (0.65)	416 (0.65)	10 (1.36)	.02
Antihypertensive drugs	824 (1.27)	811 (1.26)	13 (1.77)	.22
Antidiabetic drugs	751 (1.15)	729 (1.13)	22 (2.99)	<.001
NSAIDs^f^	2440 (3.75)	2406 (3.74)	34 (4.63)	.21
Health care utilization, n (%)				
Hospitalization	8033 (12.34)	7935 (12.33)	98 (13.33)	.41
Number of distinct prescription drugs (≥1)	48,386 (74.35)	47,827 (74.33)	559 (76.05)	.29
Pregnancy characteristics^b^, n (%)				
Gestational duration (weeks), mean (SD)	38.99 (1.44)	39.01 (1.41)	37.33 (3)	<.001
Multiple pregnancy	877 (1.35)	846 (1.31)	31 (4.22)	<.001
Parity				<.001
Nulliparous	29,284 (44.99)	28,888 (44.89)	384 (52.24)	
Others	35,799 (55.01)	35,460 (55.11)	351 (47.76)	
Secondary outcomes, n (%)				
Low Apgar score at 5 minutes	305 (0.47)	205 (0.32)	100 (13.61)	<.001
Complete adherence to ANC	41,317 (63.48)	40,947 (63.63)	370 (50.34)	<.001
Antenatal maternal care^g^				
Appropriateness of gynecological visits	58,341 (89.64)	57,709 (89.68)	632 (85.99)	.001
Promptness of gynecological visits	61,338 (94.25)	60,650 (94.25)	688 (93.61)	.45
Appropriateness of ultrasound examinations	60,997 (93.72)	60,317 (93.74)	680 (95.52)	.18
Appropriateness of laboratory tests, n (%)				<.001
Complete adherence	46,718 (71.78)	46,298 (71.95)	420 (57.14)	
Partial adherence	16,622 (25.54)	16,353 (25.41)	269 (36.60)	
Nonadherence	1743 (2.68)	1697 (2.64)	46 (6.26)	

a *t* test, Chi-square, or Fisher exact test, as appropriate.

b Data related to the current pregnancy.

c ≥14: at least bachelor’s degree; 6‐13: lower or upper secondary education; ≤5: primary education or none.

d Measured from one year before pregnancy through 22 weeks of gestation.

e Included diagnoses of migraine/headache, epilepsy, bipolar disorder, personality disorder, other psychiatric disorders, psychosis or schizophrenia, and sleep disorder.

f NSAID: nonsteroidal anti-inflammatory drug.

g ANC: antenatal care.

**Figure 2.**
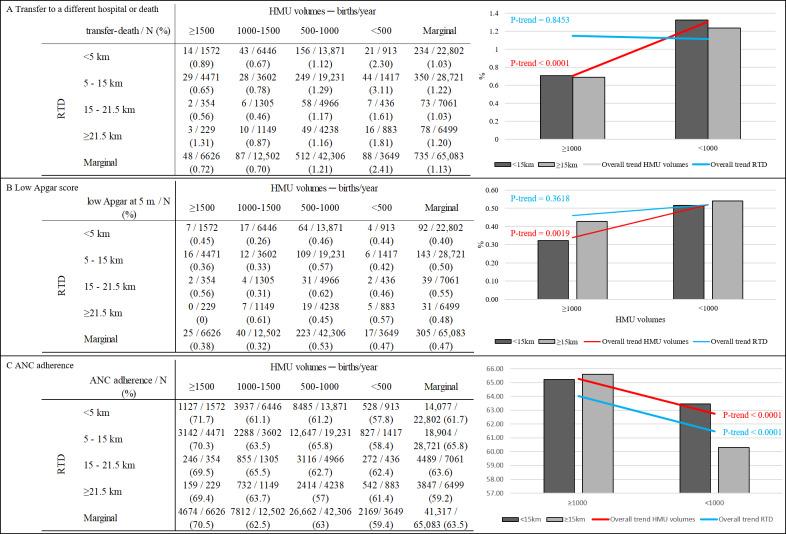
Distribution of maternal and neonatal outcomes based on HMU volume and RTD in Lombardy (N=65,083). (A) Transfer to a different hospital or death; (B) low Apgar score; (C) ANC adherence. ANC: antenatal care; HMU: hospital maternity unit; RTD: road travel distance.

Figure 2 illustrates the distribution of outcomes by HMU volume and RTD. The rate of the primary outcome (transfer or death) significantly increased with decreasing HMU volume (*P* trend<.001). Hospitals with fewer than 500 births/year had a rate of 2.41%, compared with 0.72% in hospitals with 1500 births/year (Figure 2A). No significant differences were observed for RTD (*P* trend=.84). However, the lowest rate of the primary outcome was consistently observed in births with both high HMU volumes (≥1000 births/year) and short RTD (<15 km), representing the “best scenario” compared with all other combinations of volume and distance. Although too uncommon for detailed analysis across all categories, low Apgar scores were more prevalent in low HMUs with longer RTD (*P* trend<.002) (Figure 2B). Complete adherence to ANC decreased with lower HMU volume and increased with shorter RTD (*P* trend<.001, Figure 2C).

**Figure 3.**
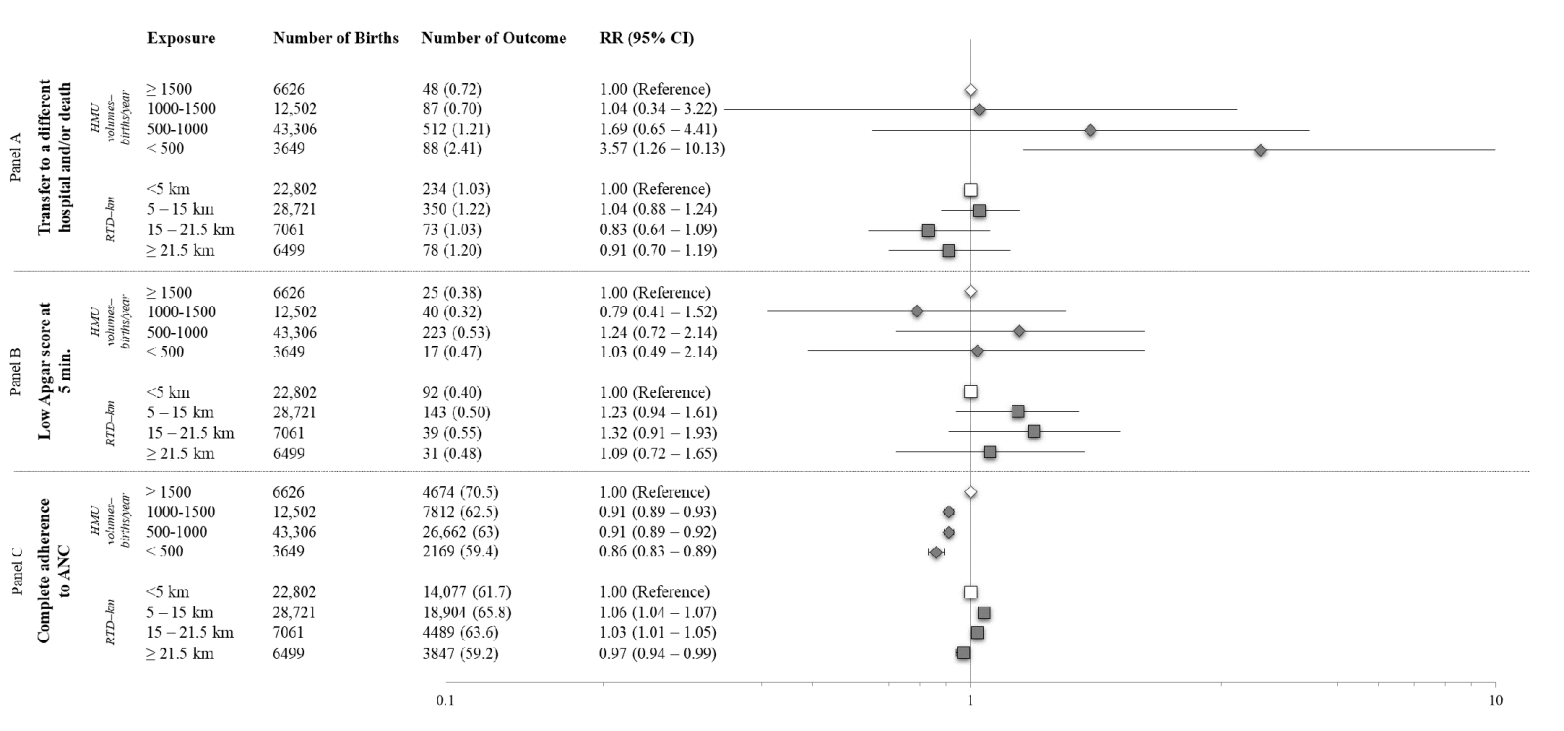
Association between HMU volumes, RTD, and primary and secondary outcomes in Lombardy in 2016‐2019 (N=65,083). (A) transfer to a different hospital or death; (B) low Apgar score; (C) ANC adherence. ANC: antenatal care; HMU: hospital maternity unit; RTD: road travel distance.

The association between low HMU volume and the primary outcome was confirmed by log-binomial regression models (Figure 3). Newborns born in maternity units with fewer than 500 births/year highlighted had up to a 3-fold higher risk of being transferred or dying compared with those delivered in hospitals with 1500 or more births/year (adjusted RR 1.69, 95% CI 0.65-4.41) and newborns born in hospitals with an HMU volume between 500‐1000 and <500 (adjusted RR 3.57, 95% CI 1.26-10.13), respectively (Figure 3A). In contrast, no statistically significant associations were observed when evaluating the RTD and its interaction with volumes while considering the low Apgar score as a secondary outcome (Figure 3B). Conversely, the RR of adhering to ANC recommendations decreased with lower HMU volume. Women who delivered in maternity units with 1000‐1500 births/year had a 9% (95% CI 7%‐11%) lower adherence rate, whereas those in units with <500 births/year had a 14% (95% CI 11%‐17%) lower adherence rate compared to high-volume hospitals. Additionally, longer RTD was associated with a 3% (95% CI 1%‐6%) lower adherence rate (Figure 3C).

**Figure 4.**
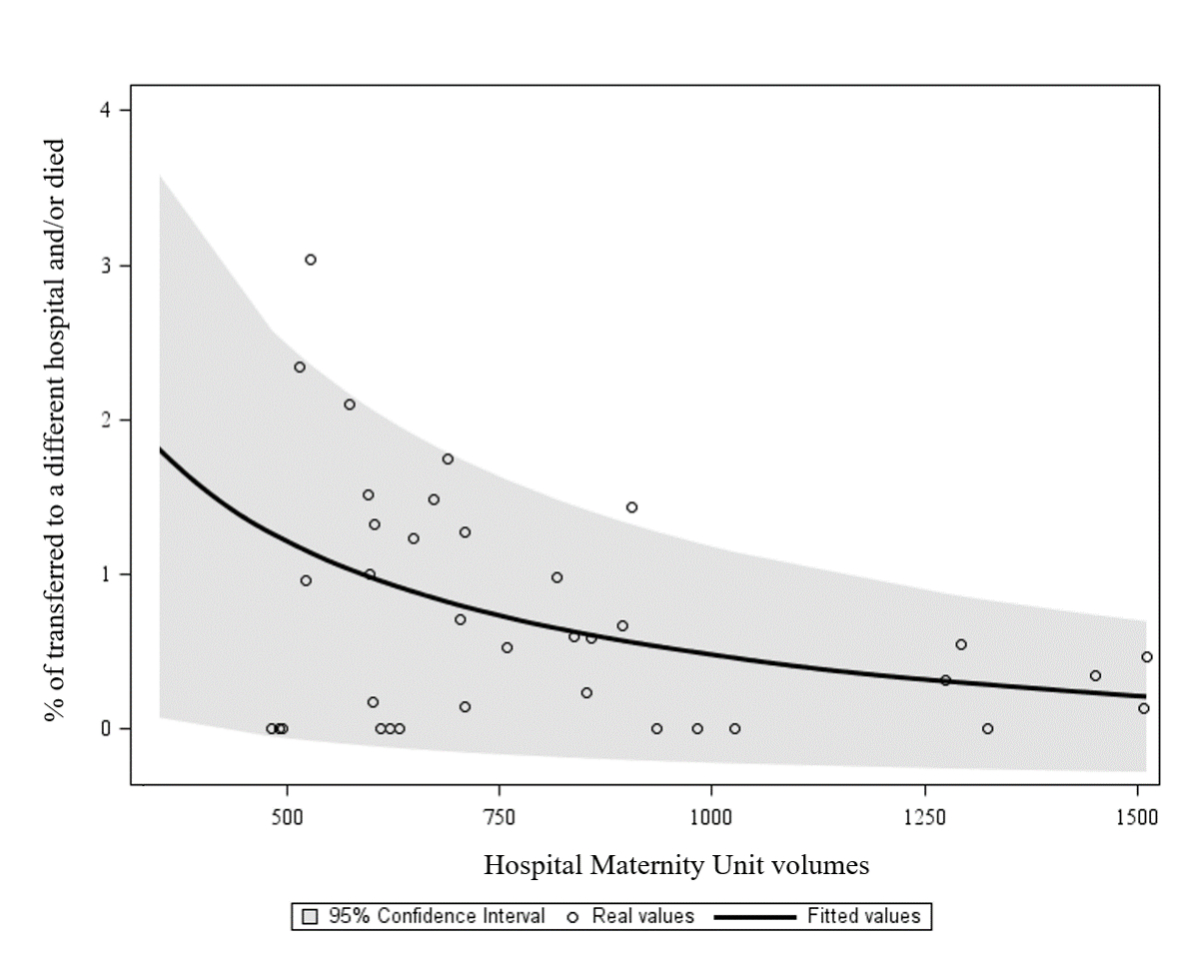
Distribution of the percentage of newborns who were transferred to a different hospital or died as a function of HMU volumes in Lombardy in 2016‐2019.

These results were confirmed through a stratified analysis by hospital birth volume (high: ≥1000 births/year, low: <1000 births/year) (data not shown). Figure 4 shows the distribution of the percentage of newborns who were transferred to a different hospital or died as a function of HMU volumes. The FPM identified was $\frac{1}{x}+\ln\left(x\right)*\frac{1}{x}$, where x represents the mean number of births in each hospital in 2019. Each point on the graph represents a hospital included in our cohort. The solid black line demonstrates a clear decreasing trend in the frequency of the primary outcome (ie, percentage of individuals transferred to a different hospital and/or died), ranging from 2% in hospitals with lower activity volumes to 0.5% in those with higher activity volumes. There is also a specific trend of the curve flattening at an HMU volume of 1000 births/year or greater, where the frequency of the primary outcome was around 0.5%.

## Discussion

Our findings showed an excess risk of both neonatal transfer and death for live births delivered in HMUs with low activity volumes, comparable to services without ICUs. In contrast, the distance between the mothers’ residences and hospital locations did not affect the primary outcome. Neither HMU volume nor RTD were significantly associated with low Apgar scores. However, mothers referred to hospitals with higher activity volumes and shorter travel distances demonstrated better adherence to ANC pathways, suggesting that being assisted by a lower-volume and distant hospital is associated with poorer ANC and correlates with a higher risk of neonatal problems. Finally, the data show a flattening trend in the prevalence of transfer to a different hospital or death in hospitals with an activity volume of 1000 births/year or more. This finding suggests this cutoff as the acceptable number of births for each maternity hospital to ensure an appropriate standard of care at delivery.

Maternity care reconfiguration is a complex and controversial issue in health care. It involves consolidating or closing small, less specialized maternity units and centralizing childbirth services at larger, more specialized hospitals. There are a number of reasons supporting the regionalization of HMUs. These include (1) declining birth rates, (2) improved safety and outcomes, and (3) saving costs.

In many countries, including Italy, the birth rate has declined for several decades. This has reduced the number of births—mainly at already small hospitals—increasing the risk of inefficient operation, low occupancy rates, and increasing maternal and neonatal adverse outcomes. However, regionalization of perinatal care could be the right solution to improve outcomes for mothers and newborns. For example, in Portugal and many other countries, regionalization involved the closure of a massive number of HMUs with fewer than 1000 births/year. This has consistently reduced several maternal and neonatal adverse outcomes such as the stillbirth rate, which ranged from 4 per 1000 births in 2000 to 2.2 per 1000 births in 2021 [17]. In contrast, in Italy, despite a decreased number of births, the stillbirth rate has remained consistent over the years, ranging from 2.4 to 2.2 per 1000 births during the same period. However, several studies have shown that women who give birth at large hospitals with high volumes of deliveries tend to have better outcomes, including lower rates of complications and infant mortality [18]. Our results are consistent with findings from other studies that reported significantly lower rates of stillbirths and neonatal mortality in both rural and urban regions after the closure of low-volume HMUs [19].

While data indicate lower neonatal mortality rates in high-volume birth hospitals at an early age, closing low-volume units requires extreme caution due to potential disadvantages, and is the subject of ongoing debate. Studies have raised concerns about increased unplanned out-of-hospital births and high neonatal mortality and stillbirth rates following closures [20,21]. Furthermore, studies in vast rural areas with limited access to perinatal care have revealed high rates of adverse birth outcomes and increased stress and anxiety among pregnant women [22,23]. However, our results did not show an association between long distances traveled to access perinatal care and the analyzed neonatal outcomes, except among hospitals with a low volume of activity and for full adherence to ANC.

Moreover, centralizing childbirth services can help to reduce health care costs by eliminating duplication of services and improving the efficient use of resources. However, there are several potential drawbacks to maternity care reconfiguration including increased travel times for women (if women must travel farther and for a longer time to give birth, this can increase their risk of complications), which may make receiving adequate prenatal care more difficult. Unfortunately, in this study, we were only able to calculate the distance to access perinatal care, which is a proxy for travel time rather than the time travel itself. Moreover, the closure of small maternity units can leave some women with limited access to childbirth services, especially in rural or geographically disadvantaged areas. It must be considered that access to ANC is often guaranteed in nonhospital settings (eg, public clinics) and that low-level hospitals can offer adequate ANC while organizing a structured referral to large hospitals for delivery. High-performing hospitals and well-equipped neonatal ICUs can also manage critical situations without the need to transfer newborns.

The decision to regionalize perinatal care units is complex and must be made on a case-by-case basis, considering all relevant factors. There is no one-size-fits-all solution that would be suitable for all communities, and health care providers and policy makers should consider several factors before making decisions about maternity care reconfiguration. These include the specific needs of the community, availability of transportation, and access to specialized care.

A strength of our study is the large, population-based sample reporting real-world evidence on the associations between HMU volumes and RTD, and the transfer to a different hospital or death (identified through a robust definition), low Apgar score at 5 minutes, and complete adherence to ANC. Moreover, we considered the individual prenatal care pathway for each mother, strengthening our analysis by addressing a potential confounding factor.

A limitation of our study is the lack of data on the actual time to travel to HMUs, as only data on RTD were available; we acknowledge that factors such as traffic congestion, mode of transportation, and individual driving habits could influence actual travel times. In addition, due to privacy concerns, we lack data on the exact location of the mothers’ residence; therefore, we used the mothers’ residence municipality for the calculation of the RTD. This misclassification might have reduced the difference in outcomes between RTD categories, thus underestimating the effect of RTD on outcomes. However, we believe that the RTD data used in our analysis provides a valuable approximation of accessibility, particularly when considered in conjunction with other factors such as HMU volumes and neonatal outcomes. Moreover, we were not able to consider several other factors, such as smoking, alcohol, illicit drug use, and social factors related to migration status that may affect access to both ANC and the HMU and influence the outcomes of interest. Additionally, the exclusion of mothers who experienced a stillbirth or those with missing information for sociodemographic or clinical characteristics likely affected less healthy women. However, missing data were infrequent (at most 0.4% for employment status). Nevertheless, outcome prevalence did not vary between complete and incomplete cases, supporting the hypothesis of data “missing at random” (data not shown). In our previous study, we showed that multiple imputations did not change the complete-case analysis results [24]. Finally, our results are derived from Lombardy, a specific Italian region, which may impact the generalizability of results. However, we believe that our study’s key insights can apply to a broader range of settings, particularly those with similar health care systems and demographic characteristics. However, cultural beliefs and preferences regarding childbirth and health care can affect the decision-making process for mothers and health care providers. While our study provides valuable insights into the relationship between HMU volume, RTD, and neonatal outcomes in Lombardy, it is important to consider these factors when applying the findings to other regions. Several studies have reported that regionalized care enhances patient outcomes by concentrating specialized expertise at high-volume centers and fostering collaboration among health care providers within a defined region [25]. Moreover, studies have consistently demonstrated the benefits of regionalized care for high-risk infants, particularly those born very or late preterm and very-low-birth weight (<32 weeks of gestation) [26,27]. Further research may be needed to assess the generalizability of our results in diverse settings.

In conclusion, our results support the requirement outlined in the Italian Ministry of Health’s decree 70/2015, which mandates at least 1000 deliveries per year in HMUs as a standard. It is necessary to emphasize that maternal-newborn separation caused by the transfer of the newborn can be incredibly detrimental to their development and bonding, making neonatal transfer a major concern [28]. Therefore, it is important to have a comprehensive plan to mitigate any potential negative impacts of maternity care reorganization. This plan should include measures to ensure that women have access to appropriate prenatal care and that they are able to give birth in a safe and supportive environment, regardless of their location, as advocated by the World Health Organization.

## Abbreviations

ANC: antenatal care
FPM: fractional polynomial model
HMU: hospital maternity unit
ICU: intensive care unit
NHS: National Health Service
RR: relative risk
RTD: road travel distance
STROBE: Strengthening the Reporting of Observational Studies in Epidemiology
